# Ultra-Broadband Optical Gain in III-Nitride Digital Alloys

**DOI:** 10.1038/s41598-018-21434-6

**Published:** 2018-02-15

**Authors:** Wei Sun, Chee-Keong Tan, Jonathan J. Wierer, Nelson Tansu

**Affiliations:** 1 Center for Photonics and Nanoelectronics, Department of Electrical and Computer Engineering, Lehigh University, Bethlehem, PA 18015 USA; 2 Department of Electrical and Computer Engineering, Clarkson University, Potsdam, NY 13699 USA

## Abstract

A novel III-Nitride digital alloy (DA) with ultra-broadband optical gain is proposed. Numerical analysis shows a 50-period InN/GaN DA yields minibands that are densely quantized by numerous confined states. Interband transitions between the conduction and valence minibands create ultra-broadband optical gain spectra with bandwidths up to ~1 μm that can be tuned from the red to infrared. In addition, the ultra-broadband optical gain, bandwidth, and spectral coverage of the III-Nitride DA is very sensitive to layer thickness and other structural design parameters. This study shows the promising potential of the III-Nitride DAs with tunable ultra-broadband interband optical gain for use in semiconductor optical amplifiers and future III-Nitride photonic integration applications.

## Introduction

The tremendous demand for high efficiency light sources has driven unprecedented development of III-Nitride materials over the past decades^1–8^. Due to their direct bandgap and extremely large energy coverage (0.64 eV to 6 eV), the III-Nitride family (AlInGaN) has been widely implemented in solid-state lighting (SSL) applications^1–8^. Recently, novel applications such as visible light communication, lighting for human health, and horticultural lighting has shifted III-Nitride SSL beyond the conventional white light source to a multifunctional device^8,9^ where integration or photonic integrated circuits (PIC) are needed^8–11^. In PICs, one key component is the semiconductor optical amplifier (SOA), which is used to increase and control the light intensity within the optoelectronic circuit^10–12^.

Traditionally, III-V compound semiconductors, such as InGaAsP, have been implemented into SOA devices for telecommunication applications^10–14^. In general, the SOA in a PIC shares a similar gain medium as the laser diode, which leads to cost-efficiency and fabrication ease^10–12^. The conventional quantum nanostructures produce relatively narrow gain spectrum (<100 nm)^15–19^, limiting the integration of SOAs with multiple devices working in different spectral regimes. Several active region strategies have been proposed to increase the broadband optical transition in traditional III-V compounds, including superlattices with bound-to-continuum transitions and inter-subband transitions^20–22^, quantum dots^23–25^, and non-identical multiple quantum wells (QW)^26^. Despite of the advances of those III-V based SOAs, integrating traditional III-V compounds with III-Nitride optoelectronic devices would be difficult because of the fundamental differences in crystal structure and lattice mismatch. Thus, if the promise of III-Nitride SOAs is very compelling, it may induce new strategies to create PICs made entirely of III-Nitride semiconductors.

There have been several foundational works that can be leveraged to understand and create III-Nitride digital alloys (DAs)^27,28^ and superlattices^29–32^. The key behind the concept of III-Nitride DAs^27,28^ is to employ the ultrashort period superlattices for accessing nano-engineered materials with electronic and optoelectronics properties mimicking those of alloyed systems, which also results in improved device functionality in the form of ultra-high electron-hole wavefunction overlap. In addition, there are several experimental reports of III-Nitride short-period superlattices, which show the feasibility of applying III-Nitride DA as active regions using state-of-the-art epitaxy techniques^33–41^.

In this letter, ultra-broadband interband optical gain is analyzed and shown as a unique functionality of III-Nitride DA active regions. Our studies show the finite-period InN/GaN DA with ultra-thin barriers and quantum wells results in densely quantized minibands, which leads to ultra-broadband gain profile that is not attainable using traditional quantum wells. Our finding shows that ultra-broadband optical gain spectra with bandwidths up to ~1 μm in the red to infrared spectral range as possible. Additionally, the optical gain spectrum of the InN/GaN DA can be tuned by engineering the thicknesses of the barriers and quantum wells. Such III-Nitride DA active regions could be useful in applications such as SOAs.

## Concept and Modeling

**Figure 1:**
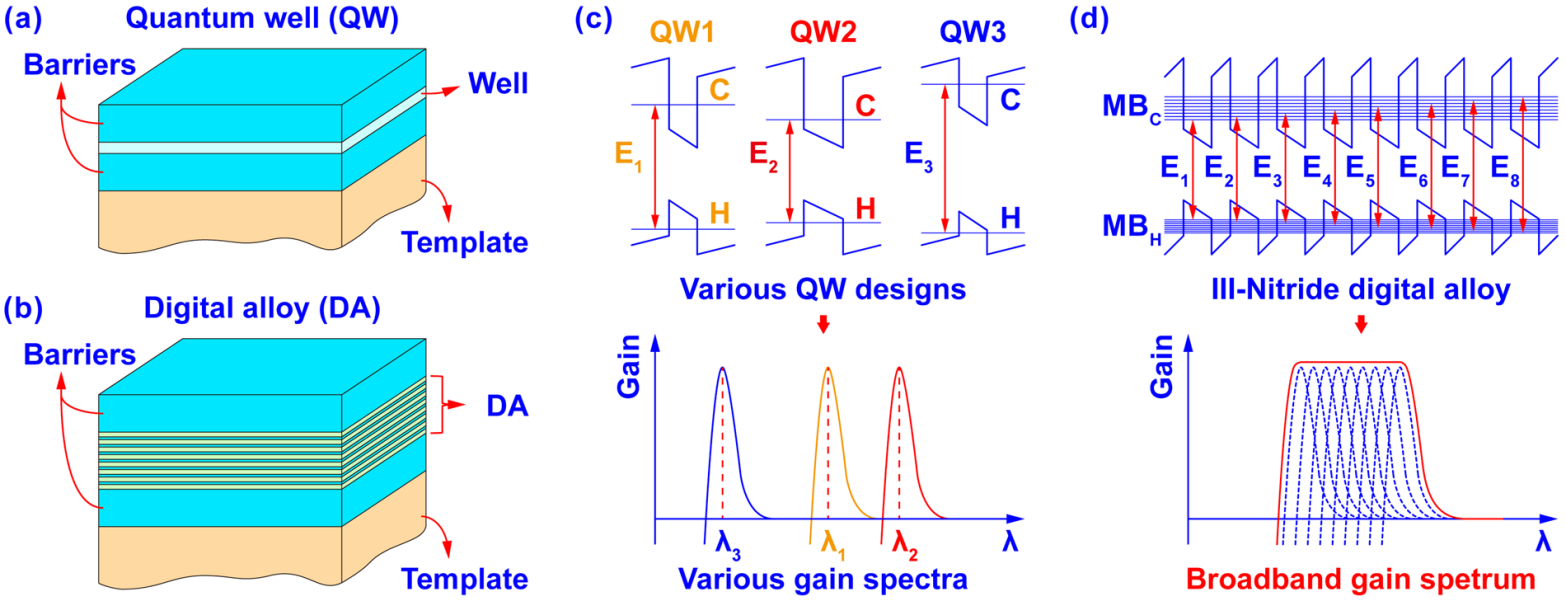
Schematic illustration of **(a)** a quantum well active region and **(b)** a digital alloy active region. **(c)** Energy band lineup of individual quantum wells with different thickness and bandgap produce optical gain spectra that are spectrally separate with narrow bandwidth. **(d)** The energy band diagrams of a digital alloy active region with quantized minibands yields broadband optical gain spectrum produced by closely packed (in energy) interband transitions.

Figure 1(a) shows a schematic of a conventional III-nitride QW active region formed by sandwiching narrow bandgap quantum wells with wider bandgap barriers. Due to quantum confinement, carriers are confined at certain energy states within the QW. The allowed interband transition between the quantum confined states produces optical gain at a sufficient carrier population. As shown in Fig. 1(c), various QW designs can be applied to produce optical transitions with different transition energies, which in turn yield various optical gain spectra at different energies. However, the bandwidth of these individual QWs’ optical gain spectra are still relatively narrow^15–19^, which restricts the implementation of QWs into SOAs.

In contrast to the QW design, the III-Nitride DA consists of an ultra-short period superlattice formed by stacking ultra-thin III-Nitride epilayers periodically^27,28^, as shown in Fig. 1(b). The use of barriers and quantum wells that are thinner than 4 monolayers (MLs) introduces strong resonant coupling of the quantum states within the DA structure. The carrier wavefunction spreads into adjacent QWs allowing the carriers to tunnel through the barriers and couple with one another. The result is the original quantum confined-states split and form minibands.

In III-Nitride DAs with finite-periods, the minibands are densely quantized by numerous confined states. As shown in Fig. 1(d), an 8-periods III-Nitride DA has a conduction miniband (MB_C_) and valence miniband (MB_H_) that both split into 8 confined states. In principle, this III-Nitride DA has 8 major interband optical transitions from the MB_C_ to the MB_H_ that form the new broadband spectrum (neglecting the plurality of hole bands). Note that these confined states are closely packed within each miniband with tiny energy difference, leading to closely packed interband transitions within the new spectrum. On the other hand, the difference between the lowest transition energy (E_1_) and the highest transition energy (E_8_) is sufficiently large, and results in the broadband spectral coverage. Furthermore, built-in electric fields due to piezoelectric and spontaneous polarization in III-Nitrides breaks the symmetry of carrier wavefunctions and promotes more possible transitions between quantum states, which in turn also contribute to the broadband spectrum.

To characterize the broadband optical gain of the InN/GaN DA structure, we carry out numerical calculations. We first model the energy band diagrams of the DA based on a modified Kronig-Penney model, in which the strain and built-in polarization fields are considered^42–46^. In our study, we assume the InN/GaN DA is grown on GaN substrates, where all InN layers within DA are compressively strained to GaN. The band structure, quantum confined states, and carrier wavefunctions of InN/GaN DA are calculated using the transfer matrix method^47^. Lastly, the interband optical gain properties of the InN/GaN DA are calculated by applying Fermi’s golden rule^42^. The optical gain is an intrinsic property of the DA active region. Thus, the non-radiative recombination processes are only considered in the total recombination current density calculations. In this letter, the bandwidth of the optical gain spectrum is defined as its full width at half-maximum (*B*_*FWHM*_). All material parameters used in this numerical study are obtained from previous reports^48,49^.

## Results and Discussion

**Figure 2:**
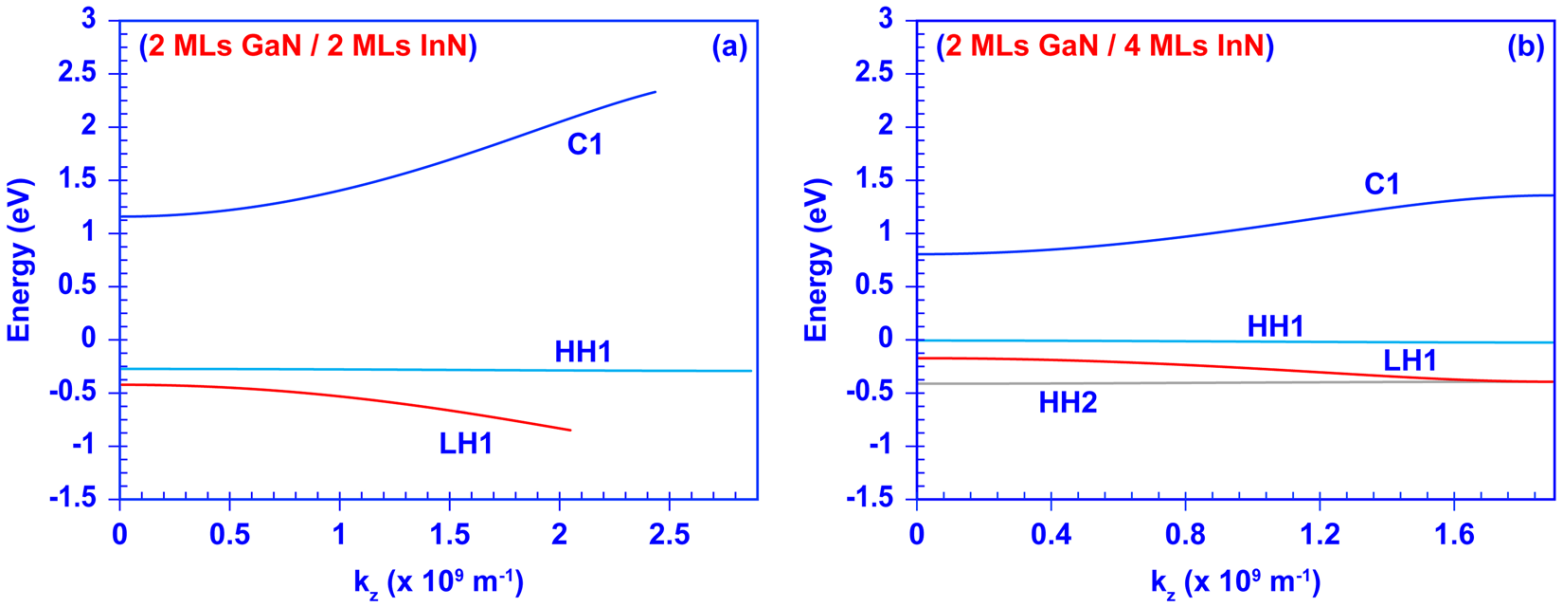
Plot of energy versus wave vector for **(a)** the InN/GaN DA formed by 2 MLs thick GaN barriers and 2 MLs thick InN quantum wells, and **(b)** the InN/GaN DA formed by 2 MLs thick GaN barriers and 4 MLs thick InN quantum wells. Note that in the finite-period InN/GaN DA all minibands are quantized into finite quantum confined states.

Figure 2 shows the calculated energy versus wave vector of two different InN/GaN DAs. The C1, HH1, and LH1 denote the ground-state conduction miniband, heavy-hole miniband, and light-hole miniband, respectively, while the HH2 is the second heavy-hole miniband. The general discussion of miniband engineering in III-Nitride DAs as function of the layer thicknesses has been presented in our previous studies^27,28^. The energy “width” of the miniband is determined by the span of the energy versus wave vector. As shown in Fig. 2(a), the DA structure with 2 MLs thick GaN barriers and InN quantum wells has sufficient inter-well resonant coupling, leading to wide minibands with widths of ~1.16 eV, ~0.03 eV, and ~0.42 eV for the C1, HH1, and LH1 minibands, respectively. In comparison, Fig. 2(b) shows the minibands of the InN/GaN DA formed by 2 MLs thick GaN barriers and 4 MLs thick InN quantum wells. By increasing the InN thickness from 2 MLs to 4 MLs, the minibands become narrower with the widths of ~0.54 eV, ~0.005 eV, and ~0.23 eV for C1, HH1, and LH1 bands, respectively. Additionally, the thicker InN quantum well has significantly lower energy levels for the minibands, leading to a redshift of the effective bandgap.

The continuous minibands of a DA with finite periods are densely quantized into numerous confined states. As we increase the periods, the number of these states become larger, which in turn force the states to move closer. For the examples shown below we calculate with 50 periods. Ideally, all possible interband transitions between these densely-packed confined states contribute to the formation of the broadband optical gain spectrum. However, the carrier density within the minibands also needs to be considered. For example, with thinner InN quantum wells the quantum confinement is strong, and results in the high energy minibands. When the carrier density in the active region is restricted, only a limited number of confined states within the high energy minibands will be populated with carriers, resulting in a limited bandwidth in optical gain spectrum. In contrast, it will be easier to populate confined states with restricted carrier densities within DAs having thicker InN quantum wells with lower energy minibands. Therefore, the final optical gain spectrum can be sufficiently broadband in a DA with thicker InN quantum wells, despite that the bandwidth of minibands are suppressed due to the weaker resonant coupling.

**Figure 3:**
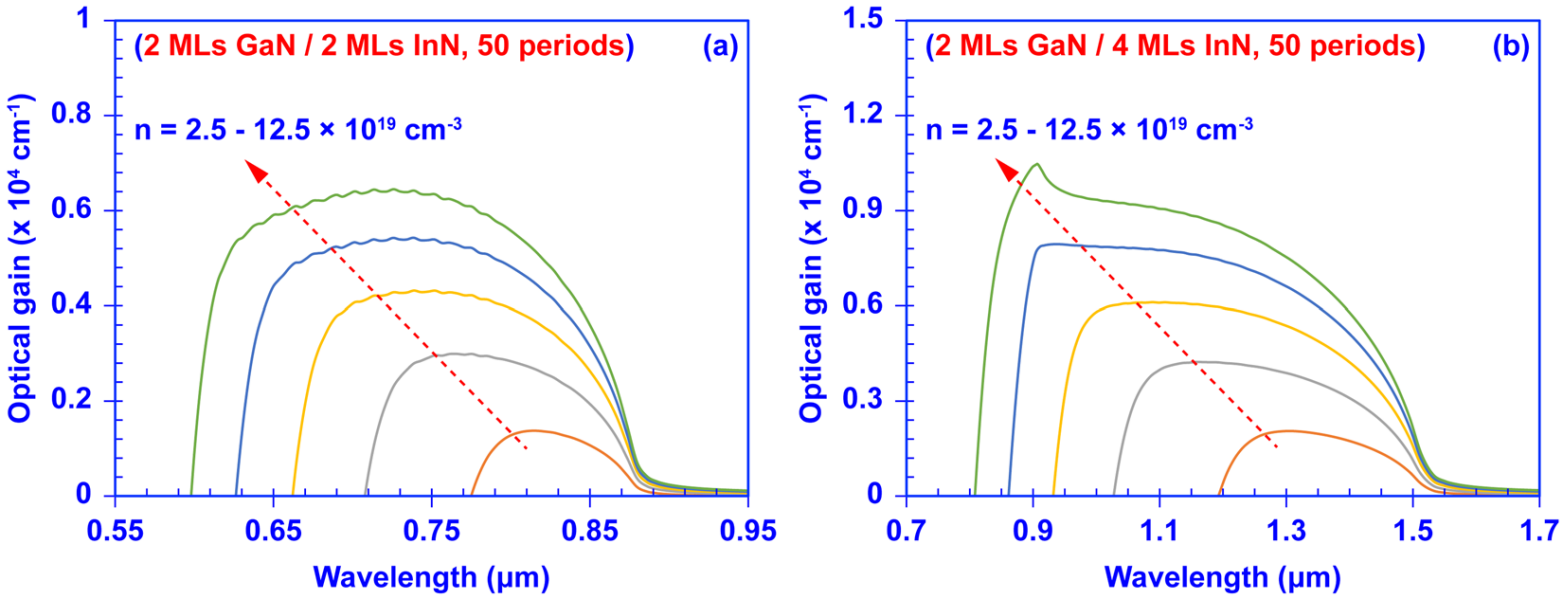
Ultra-broadband optical gain spectra of a 50-period InN/GaN digital alloy formed by **(a)** 2 MLs thick GaN barriers and 2 MLs thick InN quantum wells, and **(b)** 2 MLs thick GaN barriers and 4 MLs thick InN quantum wells. The carrier density (n) increases from 2.5 × 10^19^ cm^−3^ to 12.5 × 10^19^ cm^−3^ with increments of 2.5 × 10^19^ cm^−3^.

Figure 3 shows the calculated ultra-broadband optical gain spectra of two 50-period (p) InN/GaN DA structures at carrier densities ranging from *n* = 2.5 × 10^19^ cm^−3^ to 12.5 × 10^19^ cm^−3^. The optical gain is dominated by TE polarized light. Note the calculation treats the DA structure as one active region, and assumes uniformly distributed and steady-state carrier concentrations as initial condition. The 50-period DA formed by 2 MLs of GaN and 2 MLs of InN has a transparency carrier density of *n*_*tr*_ = 0.7 × 10^19^ cm^−3^. As shown in Fig. 3(a), as the carrier density increases from 2.5 × 10^19^ cm^−3^ up to 12.5 × 10^19^ cm^−3^, the confined states are increasingly populated with carriers, leading to significant broadening of the interband optical gain spectrum. When the carrier density reaches *n* = 10 × 10^19^ cm^−3^, the optical gain spectrum has a FWHM bandwidth (*B*_*FWHM*_) of ~225 nm. Note that the energy gap of the InN/GaN DA structure shown in Fig. 3(a) is ~1.41 eV corresponding to a cutoff wavelength of ~880 nm. Therefore, the InN/GaN DA with 2 MLs GaN and InN layers exhibits remarkably broadband optical gain over the red and near infrared regime from ~635 nm to ~860 nm.

Furthermore, increasing the thickness of the InN quantum well redshifts the bandgap of the InN/GaN DA to 0.82 eV, which extends the optical gain spectrum deeper into the infrared. As shown in Fig. 3(b), the optical gain of the 50-periods InGaN DA with 2 MLs of GaN and 4 MLs of InN begins to exhibit gain at *n*_*tr*_ = 0.6 × 10^19^ cm^−3^. The *B*_*FWHM*_ of the optical gain spectrum increases to ~580 nm when the carrier density reaches 10 × 10^19^ cm^−3^. The ultra-broadband optical gain from the InN/GaN DA covers from ~880 nm to ~1460 nm, significantly surpassing SOA active regions formed from traditional III-V compounds. Such a wide gain spectrum shows the remarkable potential of the III-Nitride DA as a SOA device. In addition, at sufficiently high carrier density, the density of states of the minibands determines the shape of the optical gain spectrum. As shown in Fig. 3(b), the optical gain spectrum exhibits an extra peak at shorter wavelengths (higher energies) when the carrier density is further increased to 12.5 × 10^19^ cm^−3^. Although the *B*_*FWHM*_ increases to ~600 nm, the peak within the gain spectrum might not be desired for certain applications requiring uniform output at various emission wavelengths. Thus, choosing the proper carrier density for operation is essential to achieve a nearly constant and ultra-broadband gain spectrum.

**Figure 4:**
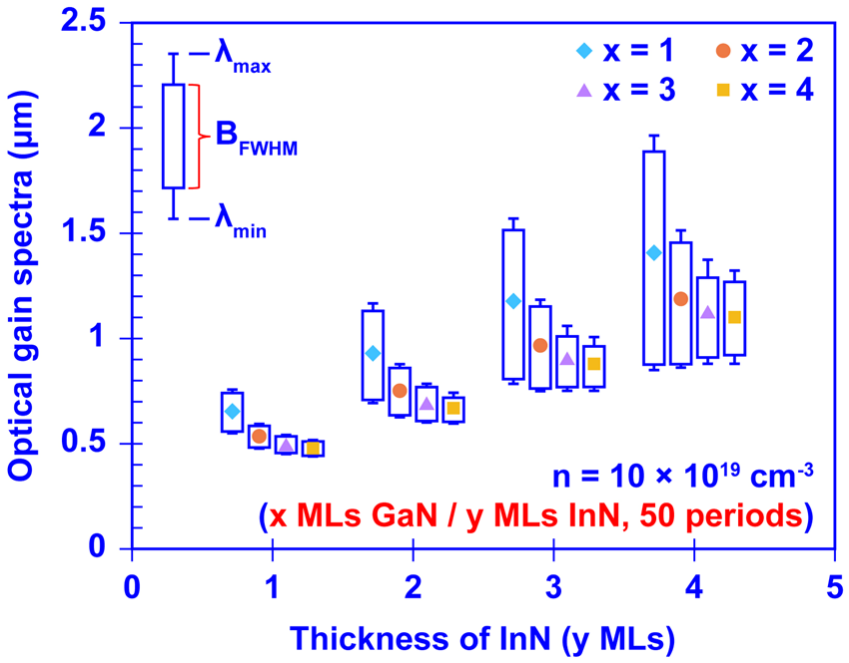
The total wavelength coverage (λ_Min_ to λ_Max_) and the bandwidth B_FWHM_ of the interband optical gain spectra of the InN/GaN DA formed by x MLs GaN barriers and y MLs InN wells at n = 10 × 10^19^ cm^−3^. The thicknesses of x and y are varied from 1, 2, 3, and 4 MLs.

To show a general picture on how the structural design of the InN/GaN DAs affects the ultra-broadband optical gain properties, we summarize the gain spectra of 16 different InN/GaN DAs in Fig. 4. The gain spectrum is presented as a vertical bar. The top and bottom segments represent the maximum wavelength (*λ*_*max*_) and minimum wavelength (*λ*_*min*_) of the optical gain spectrum respectively, while the rectangle inside denotes the *B*_*FWHM*_. Note that the *λ*_*max*_ also represents the cut-off wavelength of the optical gain spectrum. The 16 InN/GaN DAs are categorized into 4 groups based on the InN quantum well thicknesses. In each group, the thickness of GaN barriers varies from 1 ML to 4 MLs.

As shown in Fig. 4, utilizing an ultra-thin GaN barrier (1 ML) results in superior optical gain spectra with *B*_*FWHM*_ up to ~1 μm. Comparing to the thicker GaN barrier (4 MLs), the 1 ML GaN barrier brings ~2 to 3 times broader *B*_*FWHM*_ attributed to the strong resonant coupling of confined quantum states. As the GaN layers become thicker, the resonant coupling is weaker, and leads to narrower minibands and therefore smaller *B*_*FWHM*_. Meanwhile, increasing the InN well thickness also significantly increases the *B*_*FWHM*_ of the optical gain spectrum. As shown in Fig. 4, with 1 ML GaN barriers the *B*_*FWHM*_ increases from ~ 200 nm to ~1 μm when the thickness of InN increases from 1 ML to 4 MLs. As discussed previously, when the InN well becomes thicker the minibands will have lower energy. Thus, more of the confined states within low energy miniband can be populated at restricted carrier densities leading to enhanced *B*_*FWHM*_. In addition, the ultra-broadband coverage of the optical gain spectrum varies through the red to infrared wavelengths, and is a function of the GaN and InN layer thicknesses. This shows the wide range of tunability of the effective bandgap in the DA structure. Figure 4 shows that the structural design of the DA structure is very important in order to obtain the ultra-broadband optical gain spectrum with the desired spectral coverage.

**Figure 5:**
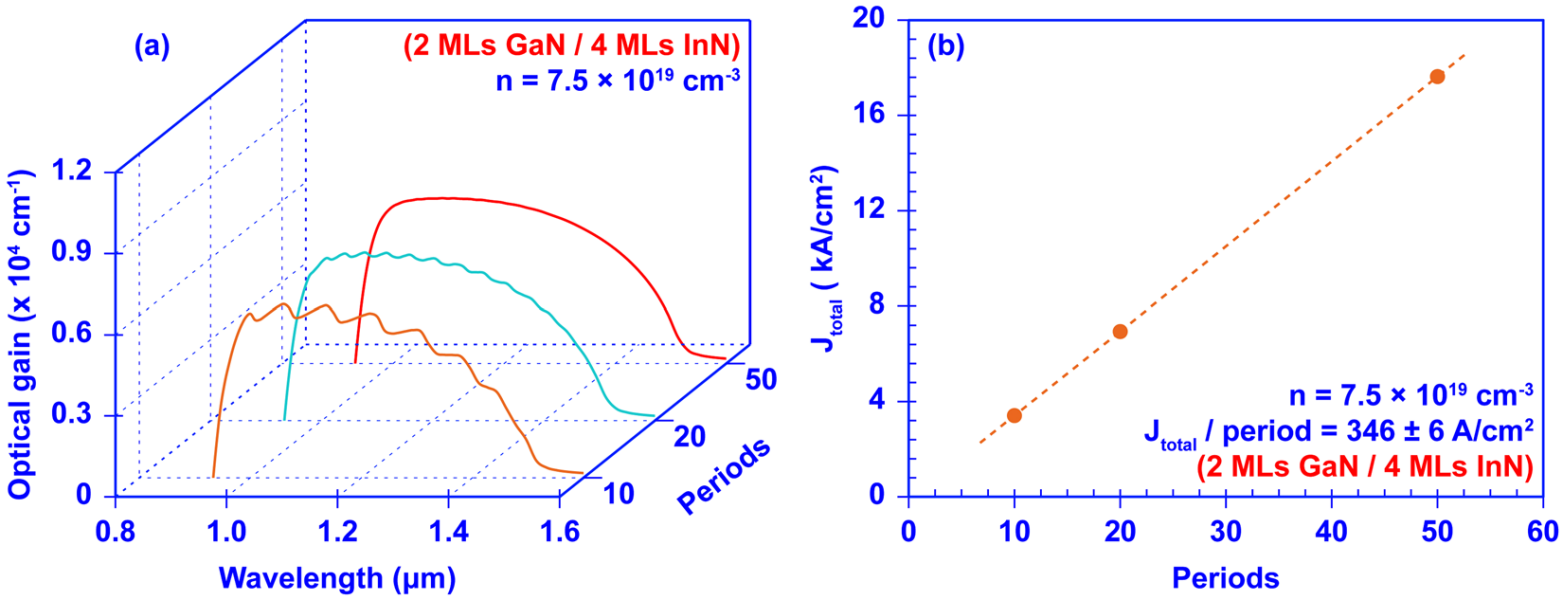
**(a)** The optical gain spectra of the InN/GaN DA structures with 50, 20, and 10 periods, respectively; and **(b)** total recombination current density J_total_ as function of periods of InN/GaN DA structures. The InN/GaN DAs consists of 2MLs thick GaN barriers with 4 MLs thick InN wells, and is operated at a carrier density (n) of 7.5 × 10^19^ cm^−3^. In **(b)** the dots are calculated and the dash line is for J_total_/period of 346 A/cm^2^.

To begin to understand the benefits and trade-offs of using InN/GaN DAs in device applications, the optical gain spectra and total recombination current density (*J*_*total*_) is calculated while varying the number of periods. Figure 5 shows the optical gain spectra and *J*_*total*_ of InN/GaN DAs with 2 MLs thick GaN barriers and 4 MLs thick InN wells with 10, 20, and 50 periods, operated at a carrier density of *n* = 7.5 × 10^19^ cm^−3^. In the calculation of *J*_*total*_, a monomolecular recombination coefficient of *A* = 1 × 10^7^ s^−1^ and an Auger recombination coefficient of *C* = 1 × 10^−34^ cm^6^ s^−3^ are assumed^50,51^. (Note: A low Auger coefficient is used here because the exact value is unknown. Determining the proper Auger coefficient for InN and InN/GaN DAs is in the early stages for this material system^52^. Clearly, a larger Auger coefficient will increase the current density). By reducing the number of periods of the InN/GaN DA from 50 to 10, the optical gain spectra change slightly. The average maximum gain remains the same at ~6000 cm^−1^, and the *B*_*FWHM*_ values range from ~517 nm to ~496 nm. The only significant compromise here at the lower number of periods is that the optical gain spectrum fluctuates slightly. The *J*_*total*_ per period is ~353 A/cm^2^, 348 A/cm^2^, and 340 A/cm^2^ for the 50, 20, and 10 period DAs, respectively. The variation of *J*_*total*_ per period is only ~2%, exhibiting a high degree of linearity between the *J*_*total*_ and the number of periods. The calculation indicates that the InN/GaN DA active region can operate at *J*_*total*_ = 3.4 kA/cm^2^ and 6.95 kA/cm^2^ with 10 and 20 periods respectively as a SOA or as an ultra-broadband laser device.

This analysis shows the trade-off for the InN/GaN DA is in the consistency (no fluctuations) of the optical gain over its bandwidth and the operating current density. The current density within the DA active region can be reduced by simply cutting down the number of periods in the DA without largely affecting the ultra-broadband optical gain. However, if consistent optical gain over the gain bandwidth is required then the DA requires a sufficient number of periods and current density. The proper structural design of the III-Nitride DA active region ultimately will be determined by the application requirements and this analysis provides some initial insight into the design trade-offs.

Our study theoretically shows that ultra-broadband interband optical gain can be achieved with a III-Nitride DA. Currently, creating the III-Nitride DA (with layer thicknesses ≤4 MLs) is still challenging using state-of-the-art semiconductor growth techniques, especially for the case of InN/GaN superlattices. Specifically, recent experimental studies on growing monolayer InN/GaN superlattices show that the samples actually have InGaN layers (with In-content of up to ~0.33) instead of InN layers, which indicates the limited In-incorporation in the InN/GaN superlattices^53,54^. Our goal of this study is: (1) to show the unique functionality of ultra-broadband optical gain of III-Nitride DAs, and (2) to provide motivation to develop growth techniques that can create the proposed DA structure. One possible first experimental demonstration could be carried out using more conventional GaN/InGaN heterostructures that target the visible spectral regime. Also, further theoretical work is necessary in order to implement III-Nitride DAs into devices. For example, implementing III-nitride DAs into SOA structures requires further studies to better understand the carrier transport, carrier distribution, and modal gain properties when including optical waveguide designs.

## Conclusion

In conclusion, we present a novel active region based on a III-Nitride DA produces ultra-broadband interband optical gain. Our calculations show that the finite-period InN/GaN DA introduces densely quantized minibands, which can be engineered through tuning of the ultra-thin barriers and quantum wells. With sufficient carrier population, the interband transitions between the minibands quantized confine-states result in ultra-broadband optical gain spectra with B_FWHM_ up to ~1 μm in across the red and infrared spectral regime. In addition, our study also presents the importance of tailoring the structural design of the DA structure in order to achieve the ultra-broadband optical gain at the desired bandwidth and spectral coverage. Our study shows the promising potential of the III-Nitride DA active region with tunable ultra-broadband interband optical gain for use in SOA devices and III-Nitride photonic integration applications.
